# A Computationally Efficient Labeled Multi-Bernoulli Smoother for Multi-Target Tracking †

**DOI:** 10.3390/s19194226

**Published:** 2019-09-28

**Authors:** Rang Liu, Hongqi Fan, Tiancheng Li, Huaitie Xiao

**Affiliations:** 1 National Key Laboratory of Science and Technology on ATR, College of Electronic Science, National University of Defense Technology, Changsha 410073, China; liurang13@163.com (R.L.); fanhongqi@nudt.edu.cn (H.F.); 2 Key Laboratory of Information Fusion Technology (Ministry of Education), School of Automation, Northwestern Polytechnical University, Xi’an 710072, China

**Keywords:** random finite set, bayes smoother, labeled multi-Bernoulli, multi-target tracking, Sequential Monte Carlo

## Abstract

A forward–backward labeled multi-Bernoulli (LMB) smoother is proposed for multi-target tracking. The proposed smoother consists of two components corresponding to forward LMB filtering and backward LMB smoothing, respectively. The former is the standard LMB filter and the latter is proved to be closed under LMB prior. It is also shown that the proposed LMB smoother can improve both the cardinality estimation and the state estimation, and the major computational complexity is linear with the number of targets. Implementation based on the Sequential Monte Carlo method in a representative scenario has demonstrated the effectiveness and computational efficiency of the proposed smoother in comparison to existing approaches.

## 1. Introduction

Multi-target tracking (MTT) has been widely used in many engineering fields including aerospace surveillance, biomedical analytics, autonomous driving, indoor localization, robotic networks and so on [1,2,3,4,5]. In the applications, both the number of targets and their states may vary in time, and the measurements are obscured by clutter and missed detection [2,3]. Traditionally, approaches to MTT are built on the base of appropriate data association methods such as typically joint probabilistic data association [1] or multiple hypothesis tracking [6,7]. A novel approach has been developed by Mahler based on the random finite set (RFS) theory [2] which has attracted substantial attention in the last decade. Simply speaking, the RFS methods model the target states and the measurements into the RFSs explicitly and have gained tremendous interest in recent years [8]. A variety of RFS filters have been proposed, including probability hypothesis density (PHD) filter [9,10], cardinalized PHD (CPHD) filter [11], Cardinality Balanced multi-Bernoulli (CBMeMBer) filter [12], generalized labeled multi-Bernoulli (GLMB) filter [13,14] and labeled multi-Bernoulli (LMB) filter [15]. Compared with the filtering that refers the current state, the smoothing [16,17,18,19] typically refers to estimating the target state of the past time using all measurements till the current time which has a better accuracy than filtering but also suffers from a higher computational cost. Therefore, it is of great significance in practice to develop an RFS smoother that is computationally efficient, reliable and accurate.

The Bernoulli smoother was first proposed in [20,21]; it performs better than the Bernoulli filter, but adapts to, at most, one target. For the multi-target problem, the forward–backward PHD smoothers are proposed in [17,18,22,23,24]. It has been shown in [17] that the PHD smoother can improve the accuracy of position estimation as compared with the PHD filter, but does not necessarily gain better cardinality estimation. The CPHD smoother is proposed in [25] which uses an approximate scheme to overcome the intractability of the classic CPHD smoother [26] but bears a complicated algorithm structure. A forward–backward multi-target multi-Bernoulli (MeMBer) smoother is proposed in [27] which also does not necessarily improve the cardinality estimation. However, these smoothers, as well as the corresponding filters, do not provide the information for each track. Therefore, labelled RFS-based filters and smoothers have been developed [28,29,30,31,32], for generating track estimates which is also the focus of this paper.

The GLMB smoother is proposed in [32], which is the first exact closed form solution to the smoothing recursion based on labeled RFS but has not been implemented in practice due to the overcomplicated data associations. Recently, Chen has pointed out the challenge to form tracks since the optimal solution is not as simple as labeling [33]. Relevantly, a multi-scan GLMB filter based on the smoothing-while-filtering framework is proposed in [34] for better labeling. However, the truncation of the multi-scan GLMB filter needs to solve an NP-hard multi-dimensional assignment problem. In short, these existing labeled smoothers have a high computational complexity even if it is practically implementable. Furthermore, we point out that, what is also related to the framework of smoothing-while-filtering [34] is the joint smoothing and filtering [19,35,36] based on which so far, however, only a single target is considered.

In this paper, we derive a forward–backward LMB smoother for multi-target tracking. Preliminary and limited results have been published in [37]. This paper provides additional results, complete proofs, and additional experiments. The proposed smoother consists of two parts regarding the forward LMB filtering and backward LMB smoothing, respectively. While in the former we apply the standard LMB filter [15], the key contribution of our work lies in the backward smoothing algorithm design. We prove that the proposed backward LMB smoothing is closed under the LMB prior for the standard multi-target system models and the backward smoothed density of each track is similar to the Bernoulli backward smoothed density [20]. Superior to the approximate parameteric/Gaussian (mixture) approximation [38], the Sequential Monte Carlo (SMC) method is a powerful method for representing arbitrary/non-Gaussian models [4]. Based on the SMC method, the proposed smoother reduces both the state error and the cardinality error as compared with the PHD smoother [17], the MeMBer smoother [27] and the LMB filter [15], and has a lower computational complexity as compared with the PHD smoother and the MeMBer smoother.

The rest of the paper is organized as follows. Basic definitions of labeled RFS and the multi-target Bayes forward–backward smoother are reviewed briefly in Section 2. The proposed forward–backward LMB smoother is detailed in theory in Section 3 and implemented based on the SMC in Section 4, respectively. Simulation results are presented in Section 5 before we conclude in Section 6.

## 2. Background

### 2.1. Notation

Single target states are denoted by lowercase letters, for example, *x* and x. Multi-target states are denoted by uppercase letters, for example, *X* and X. *x* and *X* are the unlabeled state representations. x and X are the labeled state representations. State spaces are denoted by blackboard bold letters, for example, a label space which contains a countable number of distinct labels is denoted as L, and a unlabeled target state space is denoted as X. A labeled single target state has the form $x=\left(x,\ell \right)$, where x∈X and ℓ∈L. A labeled multi-target state has the form $X=\left\{x_{1},...,x_{i},...,x_{\left|X\right|}\right\}$, where $x_{i}$ denotes a labeled single target state in X, $\left|\cdot \right|$ denotes the cardinality (or the number of targets) of a multi-target set, and X⊆X×L.

The projection L:X×L→L is to make $L\left(x\right)=\ell$ and $L\left(X\right)=\left\{L(x):x\in X\right\}$. Then define a generalized Kronecker delta function and inclusion function as:

(1) $$\delta_{Y}\left(X\right)≜\left\{\begin{array}{cc} 1, & X=Y;\\ 0, & \mathrm{otherwise}. \end{array}\right.$$

(2) $$1_{Y}\left(X\right)≜\left\{\begin{array}{cc} 1, & X\subset Y;\\ 0, & \mathrm{otherwise}. \end{array}\right.$$

Define $\Delta \left(X\right)≜\delta_{\left|X\right|}\left(\left|L\left(X\right)\right|\right)$. The inner product of $f\left(x\right)$ and $g\left(x\right)$ on a labeled single target space is defined as

(3) $$\langle f,g\rangle =\int f\left(x\right)g\left(x\right)dx=\int f(x)g(x)dx$$

The multi-target exponential form of X is defined as

(4) $$h^{X}≜\left\{\begin{array}{cc} 1, & X=⌀;\\ \prod_{x\in X}h\left(x\right), & X\neq ⌀. \end{array}\right.$$

### 2.2. GLMB and LMB RFS

The GLMB RFS X is a Labeled RFS constructed by labeled multi-target states. The distribution of an GLMB RFS [13] is exactly closed under the prediction and update of the multi-target Bayes filter. The GLMB distribution is denoted as

(5) $$\pi \left(X\right)=\Delta \left(X\right)\sum_{c\in C}\omega^{\left(c\right)}\left(L\left(X\right)\right){\left[p^{\left(c\right)}\right]}^{X}$$

where C is a discrete index set, $p^{\left(c\right)}\left(\cdot ,\ell \right)$ is the density of the track *ℓ*, $\omega^{\left(c\right)}\left(I\right)$ is the nonnegative weight of the hypothesis $\left(c,I\right)$, and $\sum_{I\subset L}\sum_{c\in C}\omega^{\left(c\right)}\left(I\right)=1$, $\int p^{\left(c\right)}\left(x,\ell \right)dx=1$.

The distribution of an LMB RFS with the parameter set $\pi ={\left\{\left(r^{\ell },p^{\ell }\right)\right\}}_{\ell \in L}$ is given by [15]

(6) $$\pi \left(X\right)=\Delta \left(X\right)\omega \left(L\left(X\right)\right)p^{X}$$

where

(7) $$\omega \left(I\right)=\prod_{\ell \in L}\left(1-r^{\ell }\right)\prod_{\ell \in I}\frac{1_{L}\left(\ell \right)r^{\ell }}{1-r^{\ell }}$$

(8) $$p^{\ell }≜p\left(x,\ell \right)$$

where $r^{\ell }$ denotes the existence probability of the track *ℓ*, $p\left(\cdot ,\ell \right)$ denotes the density, and $\omega \left(I\right)$ denotes the weight of the hypothesis $I=\left\{\ell_{1},...,\ell_{\left|I\right|}\right\}$. Note that L is a discrete countable space and the number of labels in L equals to the number of the Bernoulli components (with non-zero existence probability) in the LMB RFS.

An LMB RFS is a special case of an GLMB RFS, and the densities and the existence probabilities of different tracks in an LMB RFS are both uncorrelated. Two properties associated with an LMB RFS are as follows. More specifically, the cardinality distribution [2] is given by

(9) $$p\left(n\right)=\left(\prod_{\ell \in L}\left(1-r^{\ell }\right)\right)\sigma_{\left|L\right|,n}\left(\frac{r^{\ell_{1}}}{1-r^{\ell_{1}}},\cdot \cdot \cdot ,\frac{r^{\ell_{\left|L\right|}}}{1-r^{\ell_{\left|L\right|}}}\right)$$

where $\sigma_{\nu ,n}\left(x_{1},\cdot \cdot \cdot ,x_{\nu }\right)$ is the elementary homogeneous symmetric function of degree *n* in ν variables.

**Lemma** **1.** *If $X_{a}$ and $X_{b}$ are both LMB RFSs with the probability densities $\pi_{a}\left(X_{a}\right)$ and $\pi_{b}\left(X_{b}\right)$, respectively, where $X_{a}\subseteq X\times L_{a}$, $X_{b}\subseteq X\times L_{b}$ and $L_{a}⋂L_{b}=⌀$, $X=X_{a}⋃X_{b}$ is an LMB RFS with the probability density $\pi \left(X\right)$ and vice versa. The probability densities of $\pi_{a}\left(X_{a}\right)$, $\pi_{b}\left(X_{b}\right)$ and $\pi \left(X\right)$ have the relations as follows :*

(10) $$\pi \left(X\right)=\pi_{a}\left(X_{a}\right)\pi_{b}\left(X_{b}\right)$$

The proof of Lemma 1 is given in the Appendix A.

### 2.3. Multi-Target Bayes Forward–Backward Smoother

**Figure 1:**
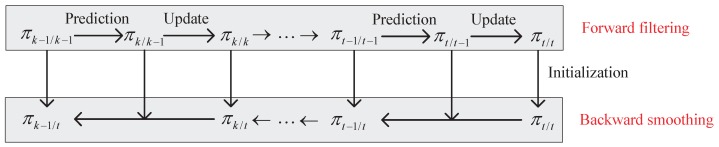
The recursion of the multi-target Bayes forward–backward smoother.

The recursion of multi-target Bayes forward–backward smoother is shown in Figure 1 which consists of forward Bayes filtering and backward Bayes smoothing. The multi-target state at *t* is $X_{t}$, where $X_{t}\subseteq X\times L_{1:t}$ and $L_{1:t}$ denotes the label space of the targets at *t* (including those born prior to *t*). The measurement set at *t* is $Z_{t}$. The prediction and update for the forward Bayes filtering can be denoted as [2]

(11) $$\pi_{t|t-1}\left(X_{t}\right)=\int f_{t|t-1}\left(X_{t}|X_{t-1}\right)\pi_{t-1|t-1}\left(X_{t-1}\right)\delta X_{t-1}$$

(12) $$\pi_{t|t}\left(X_{t}\right)=\frac{g_{t}\left(Z_{t}|X_{t}\right)\pi_{t|t-1}\left(X_{t}\right)}{\int g_{t}\left(Z_{t}|X\right)\pi_{t|t-1}\left(X\right)\delta X}$$

where $\pi_{t|t-1}$ is denoted as the predicted multi-target density from t−1 to *t*, $f_{t|t-1}$ is denoted as the multi-target Markov transition density at *t*, $\pi_{t|t}$ is denoted as the multi-target posterior density at *t*, and $g_{t}$ is denoted as the multi-target likelihood function at *t*. The integral in the equations is the set integral. If the backward smoothed density from *t* to *k* (k≤t) is denoted by $\pi_{k|t}\left(Y\right)$ which is initialized with $\pi_{t|t}\left(Y\right)$, the backward Bayes smoothed density $\pi_{k-1|t}\left(X\right)$ at k−1 can be written as [32]

(13) $$\pi_{k-1|t}\left(X\right)=\pi_{k-1|k-1}\left(X\right)\int f_{k|k-1}\left(Y|X\right)\frac{\pi_{k|t}\left(Y\right)}{\pi_{k|k-1}\left(Y\right)}\delta Y$$

### 2.4. Multi-Target Motion and Measurement Models

Let $p_{s,t|t-1}^{\ell }≜p_{s,t|t-1}\left(x,\ell \right)$ denote the surviving probability of the target $\left(x,\ell \right)$ from t−1 to *t*. $f_{t|t-1}\left(x_{+}|\left(x,\ell \right)\right)$ denotes the single target Markov transition density from t−1 to *t*. X denotes the multi-target state at t−1 while $Y^{-}$ denotes the survival multi-target state from t−1 to *t*, where $X\subseteq X\times L_{1:t-1}$ and Y⊆X. Considering the survival targets only, multi-target Markov transition density $f_{s,t|t-1}$ is [2,13]

(14) $$f_{s,t|t-1}\left(Y^{-}|X\right)=\Delta \left(Y^{-}\right)\Delta \left(X\right){\left(1-p_{s,t|t-1}^{\ell }\right)}^{X}\prod_{\ell \in L\left(Y^{-}\right)}\frac{1_{L\left(X\right)}\left(\ell \right)p_{s,t|t-1}^{\ell }f_{t|t-1}\left(y|x,\ell \right)}{\left(1-p_{s,t|t-1}^{\ell }\right)}$$

It is assumed that the newborn targets are an LMB RFS denoted as $Y^{+}$, and $Y^{+}\subseteq X\times L_{t}$, where $L_{t}$ denotes the label space of the newborn targets at *t*. $r_{B,t|t-1}^{\ell }$ denotes the birth probability of target $\left(x,\ell \right)$ at *t* and $p_{B,t|t-1}\left(y,\ell \right)$ denotes the corresponded density. The density $f_{B,t|t-1}$ of newborn targets can be denoted as [2,13]

(15) $$f_{B,t|t-1}\left(Y^{+}\right)=\Delta \left(Y^{+}\right)\prod_{\ell \in L_{t}}\left(1-r_{B,t|t-1}^{\ell }\right)\prod_{\ell \in L\left(Y^{+}\right)}\frac{1_{L_{t}}\left(\ell \right)r_{B,t|t-1}^{\ell }p_{B,t|t-1}\left(y,\ell \right)}{\left(1-r_{B,t|t-1}^{\ell }\right)}$$

The multi-target state at *t* can be denoted as $Y=Y^{+}⋃Y^{-}$ where $Y^{+}$ and $Y^{-}$ are disjoint and independent. From Lemma 1, the joint multi-target Markov transition density $f_{t|t-1}$ is written as [2,13]

(16) $$f_{t|t-1}\left(Y|X\right)=f_{B,t|t-1}\left(Y^{+}\right)f_{s,t|t-1}\left(Y^{-}|X\right)$$

Multi-target likelihood function $g_{t}\left(Z_{t}|X\right)$ is given as [2]

(17) $$g_{t}\left(Z_{t}|X\right)=e^{-\langle \kappa ,1\rangle }\kappa^{Z_{t}}{\left(1-p_{D,t}^{\ell }\right)}^{X}\sum_{\theta \in \Theta }\left(\prod_{\theta \left(\ell \right)>0}\frac{p_{D,t}^{\ell }g_{t}\left(z_{\theta \left(\ell \right)}|x,\ell \right)}{\left(1-p_{D,t}^{\ell }\right)\kappa \left(z_{\theta \left(\ell \right)}\right)}\right)$$

where $p_{D,t}^{\ell }≜p_{D,t}\left(x,\ell \right)$ denotes the detection probability of target $\left(x,\ell \right)$ at *t*. $g_{t}\left(z|x,\ell \right)$ denotes the probability that target $\left(x,\ell \right)$ generates the measurement *z* if detected. The intensity function (or the PHD) of the Poisson clutter is $\kappa \left(\cdot \right)$. The association function $\theta :L\left(X\right)\to \left\{0,1,...,\left|Z_{t}\right|\right\}$ has the property that $\theta \left(\ell_{i}\right)=\theta \left(\ell_{i^{\prime }}\right)>0$ implies $\ell_{i}=\ell_{i^{\prime }}$. Θ denotes the set of all association functions and θ∈Θ. When $\theta \left(\ell \right)=0$, the target $\left(x,\ell \right)$ is missed in detection. When $\theta \left(\ell \right)>0$, $z_{\theta \left(\ell \right)}$ denotes the measurement associated with target $\left(x,\ell \right)$. $Z_{t}/\underset{\ell \in L\left(X\right),\theta \left(\ell \right)>0}{⋃}\;z_{\theta \left(\ell \right)}$ denotes the false alarms at time *t*.

## 3. LMB Smoother

**Figure 2:**
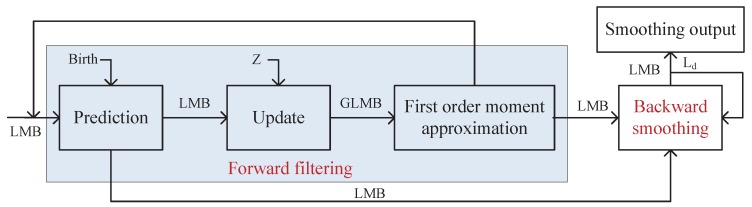
The proposed LMB smoother framework.

In this section, we detail the proposed LMB smoother and discuss the cardinality estimation. The LMB smoother framework can be simply depicted as shown in Figure 2 which consists of forward LMB filtering and backward LMB smoothing. The forward LMB filtering used in our approach is the standard LMB filter [15]. The backward LMB smoothing each time step has $L_{d}$-step backward smoothing recursions where $L_{d}$ denotes the fixed lag.

### 3.1. Forward LMB Filtering

#### 3.1.1. Prediction

Given that the multi-target prior at time t−1 is an LMB (distribution) parameterized as $\pi_{t-1|t-1}={\left\{\left(r_{t-1|t-1}^{\ell },p_{t-1|t-1}^{\ell }\right)\right\}}_{\ell \in L_{1:t-1}}$ and the density of the newborn targets is an LMB parameterized as $\pi_{B,t}={\left\{\left(r_{B,t}^{\ell },p_{B,t}^{\ell }\right)\right\}}_{\ell \in L_{t}}$, then the predicted multi-target density at *t* remains an LMB which can be denoted as [12,15]

(18) $$\pi_{t|t-1}={\left\{\left(r_{S,t|t-1}^{\ell },p_{S,t|t-1}^{\ell }\right)\right\}}_{\ell \in L_{1:t-1}}⋃{\left\{\left(r_{B,t}^{\ell },p_{B,t}^{\ell }\right)\right\}}_{\ell \in L_{t}}={\left\{\left(r_{t|t-1}^{\ell },p_{t|t-1}^{\ell }\right)\right\}}_{\ell \in L_{1:t}}$$

where

(19) $$\eta_{S,t|t-1}^{\ell }=\langle p_{s,t|t-1}^{\ell }\left(\cdot \right),p_{t-1|t-1}^{\ell }\left(\cdot \right)\rangle$$

(20) $$r_{S,t|t-1}^{\ell }=\eta_{S,t|t-1}^{\ell }r_{t-1|t-1}^{\ell }$$

(21) $$p_{S,t|t-1}\left(x_{+},\ell \right)=\frac{\langle p_{s,t|t-1}\left(\cdot ,\ell \right)f_{t|t-1}\left(x_{+}|\left(\cdot ,\ell \right)\right),p_{t-1|t-1}\left(\cdot ,\ell \right)\rangle }{\eta_{S,t|t-1}^{\ell }}$$

(22) $$p_{t|t-1}\left(x_{+},\ell \right)=1_{L_{1:t-1}}\left(\ell \right)p_{S,t|t-1}\left(x_{+},\ell \right)+1_{L_{t}}\left(\ell \right)p_{B,t}\left(x_{+},\ell \right)$$

#### 3.1.2. Update

Assume that the predicted multi-target density at *t* is an LMB with the parameter set $\pi_{t|t-1}={\left\{\left(r_{t|t-1}^{\ell },p_{t|t-1}^{\ell }\right)\right\}}_{\ell \in L_{1:t}}$, namely,

(23) $$\pi_{t|t-1}\left(X\right)=\Delta \left(X\right)\omega_{t|t-1}\left(L\left(X\right)\right){\left[p_{t|t-1}^{\ell }\right]}^{X}$$

Under the likelihood function of (17), the updated multi-target posterior density is an GLMB (Strictly speaking, it’s a δ-GLMB which is a special case of an GLMB [13]) which can be denoted as [13]

(24) $$\pi_{t|t}\left(X\right)=\Delta \left(X\right)\sum_{\left(I_{+},\theta \right)\in F\left(L_{1:t}\right)\times \Theta_{I_{+}}}\omega_{t|t}^{\left(I_{+},\theta \right)}\left(Z\right)\delta_{I_{+}}\left(L\left(X\right)\right){\left[p_{t|t}^{\theta }\left(x,\ell \right)\right]}^{X}$$

where $F\left(L_{1:t}\right)$ denotes the class of $L_{1:t}$, $I_{+}=\left\{\ell_{1},...,\ell_{\left|I_{+}\right|}\right\}$ denotes the label set of a hypothesis, $\Theta_{I_{+}}$ is the set of the association function $\theta :I_{+}\to \left\{0,1,...,\left|Z\right|\right\}$, and

(25) $$\omega_{t|t}^{\left(I_{+},\theta \right)}\propto \omega_{t|t-1}\left(I_{+}\right){\left[\eta_{Z}^{\theta }\right]}^{I_{+}}$$

(26) $$p_{t|t}^{\theta }\left(x,\ell \right)=\frac{p_{t|t-1}\left(x,\ell \right)\psi_{Z}\left(x,\ell ;\theta \right)}{\eta_{Z}^{\theta }\left(\ell \right)}$$

(27) $$\eta_{Z}^{\theta }\left(\ell \right)=\langle p_{t|t-1}\left(\cdot ,\ell \right),\psi_{Z}\left(\cdot ,\ell ;\theta \right)\rangle$$

(28) $$\psi_{Z}\left(x,\ell ;\theta \right)=\left\{\begin{array}{c} \frac{p_{D}\left(x,\ell \right)g\left(z_{\theta \left(\ell \right)}|x,\ell \right)}{\kappa \left(z_{\theta \left(\ell \right)}\right)},\theta \left(\ell \right)>0\\ 1-p_{D}\left(x,\ell \right),\theta \left(\ell \right)=0 \end{array}\right.$$

In the update of the forward LMB filtering, the multi-target posterior density matches the first-order moment of (24) for computing simplification, i.e., [15]

(29) $$\pi_{t|t}\approx {\left\{\left(r_{t|t}^{\ell },p_{t|t}^{\ell }\right)\right\}}_{\ell \in L_{1:t}}$$

where

(30) $$r_{t|t}^{\ell }=\sum_{\left(I_{+},\theta \right)\in F\left(L_{1:t}\right)\times \Theta_{I_{+}}}\omega_{t|t}^{\left(I_{+},\theta \right)}\left(Z\right)1_{I_{+}}\left(\ell \right)$$

(31) $$p_{t|t}\left(x,\ell \right)=\frac{1}{r_{t|t}^{\ell }}\sum_{\left(I_{+},\theta \right)\in F\left(L_{1:t}\right)\times \Theta_{I_{+}}}\omega_{t|t}^{\left(I_{+},\theta \right)}\left(Z\right)1_{I_{+}}\left(\ell \right)p_{t|t}^{\theta }\left(x,\ell \right)$$

The approximate posterior density (29) of the forward LMB filtering preserves the first-order moment of the posterior density (24). It is proved in [29,31] that the approximate posterior density (29) in the LMB class minimizes the Kullback-Leibler divergence (KLD) relative to the posterior density (24).

### 3.2. Backward LMB Smoothing

In this subsection, the backward LMB smoothing is derived. Our derivation directly relies on the multi-target backward smoothing recursion (13), and is different from the derivation of the backward GLMB smoothing [32] which uses the backward corrector recursion [18] as an intermediate process to avoid the set integral of the quotient of two GLMBs.

**Proposition** **1.** *Given that the multi-target posterior $\pi_{k-1|k-1}\left(X\right)$ and the predicted multi-target density $\pi_{k|k-1}\left(Y\right)$ are both LMBs, and the backward smoothed density $\pi_{k|t}\left(Y\right)$ from t to k (k≤t) is an LMB, then the backward smoothed density $\pi_{k-1|t}\left(X\right)$ from t to k−1 is also an LMB which can be written as*

(32) $$\pi_{k-1|t}={\left\{\left(r_{k-1|t}^{\ell },p_{k-1|t}^{\ell }\right)\right\}}_{\ell \in L_{1:k-1}}$$

*where*

(33) $$r_{k-1|t}^{\ell }=1-\frac{\left(1-r_{k-1|k-1}^{\ell }\right)\left(1-r_{k|t}^{\ell }\right)}{1-r_{k|k-1}^{\ell }}$$

(34) $$p_{k-1|t}\left(x,\ell \right)=\frac{p_{k-1|k-1}\left(x,\ell \right)\left(\alpha_{s,k|t}\left(x,\ell \right)+\beta_{s,k|t}\left(x,\ell \right)\int \frac{f_{k|k-1}\left(y|x,\ell \right)p_{k|t}\left(y,\ell \right)}{p_{k|k-1}\left(y,\ell \right)}dy\right)}{\int p_{k-1|k-1}\left(x,\ell \right)\left(\alpha_{s,k|t}\left(x,\ell \right)+\beta_{s,k|t}\left(x,\ell \right)\int \frac{f_{k|k-1}\left(y|x,\ell \right)p_{k|t}\left(y,\ell \right)}{p_{k|k-1}\left(y,\ell \right)}dy\right)dx}$$

(35) $$\alpha_{s,k|t}\left(x,\ell \right)≜\frac{\left(1-r_{k|t}^{\ell }\right)\left(1-p_{s,k|k-1}\left(x,\ell \right)\right)}{1-r_{k|k-1}^{\ell }}$$

(36) $$\beta_{s,k|t}\left(x,\ell \right)≜\frac{r_{k|t}^{\ell }p_{s,k|k-1}\left(x,\ell \right)}{r_{k|k-1}^{\ell }}$$

The proof of Proposition 1 is given in Appendix B. It is observed that the smoothed density $\left(r_{k-1|t}^{\ell },p_{k-1|t}^{\ell }\right)$ of the track *ℓ* has the same form as the smoothed Bernoulli density when it doesn’t take into account the newborn target [20]. Therefore, the proposed LMB smoother can be deemed as an extension of the Bernoulli smoother [20] to multiple targets. From (33), the existence probability of the track *ℓ* relates to $r_{k-1|k-1}^{\ell }$, $r_{k|k-1}^{\ell }$ and $r_{k|t}^{\ell }$. From (34), the density of the track *ℓ* contains two terms, where one term only relates to $p_{k-1|k-1}\left(x,\ell \right)$ and $\alpha_{s,k|t}\left(x,\ell \right)$ which preserves the forward filtering state at k−1, and the other term relates to the backward smoothing.

**Remark** **1.** *The proposed LMB smoother owns a good computationally efficiency to two strategies: First, the newborn tracks are uncorrelated with the backward smoothing since the newborn tracks cannot be alive prior to the birth time, resulting in a simple label space. Second, the existence probabilities and the probability densities of different tracks for an LMB RFS are uncorrelated, and then each track component can be calculated separately, so the computational complexity of the backward smoothing is linear with the number of the tracks.*

**Remark** **2.** *The proposed LMB smoother is also approximate Bayes optimal because the LMB family is closed under the prediction operation and the backward smoothing operation. Although the LMB family is not closed under the update operation, the first-order moment approximation in the LMB class minimizes the KLD relative to the posterior density.*

## 4. SMC Implementation and Algorithm Analysis

This section presents the SMC implementation and the state extraction, and analyze the algorithm complexity of the proposed LMB smoother.

### 4.1. SMC Implementation

**Prediction**: Consider the multi-target posterior at t−1 is $\pi_{t-1|t-1}={\left\{\left(r_{t-1|t-1}^{\ell },p_{t-1|t-1}^{\ell }\right)\right\}}_{\ell \in L_{1:t-1}}$. In the SMC implementation, $p_{t-1|t-1}^{\ell }$ is represented by a set of weighted particles ${\left\{\left(\omega_{t-1|t-1}^{i}\left(\ell \right),x_{t-1|t-1}^{i}\left(\ell \right)\right)\right\}}_{i=1}^{J_{t-1|t-1}^{\ell }}$. The density of the newborn targets at *t* is $\pi_{B,t}={\left\{\left(r_{B,t}^{\ell },p_{B,t}^{\ell }\right)\right\}}_{\ell \in L_{t}}$, where $p_{B,t}^{\ell }$ is also represented by a set of weighted particles ${\left\{\left(\omega_{B,t}^{i}\left(\ell \right),x_{B,t}^{i}\left(\ell \right)\right)\right\}}_{i=1}^{J_{B,t}^{\ell }}$. Through the prediction of (18)–(22), the predicted multi-target density at *t* is $\pi_{t|t-1}={\left\{\left(r_{S,t|t-1}^{\ell },p_{S,t|t-1}^{\ell }\right)\right\}}_{\ell \in L_{1:t-1}}⋃{\left\{\left(r_{B,t}^{\ell },p_{B,t}^{\ell }\right)\right\}}_{\ell \in L_{t}}$ where $r_{S,t|t-1}^{\ell }$ is calculated by (20) and $p_{S,t|t-1}^{\ell }$ is also represented by ${\left\{\left(\omega_{S,t|t-1}^{i}\left(\ell \right),x_{S,t|t-1}^{i}\left(\ell \right)\right)\right\}}_{i=1}^{J_{t-1|t-1}^{\ell }}$. That is,

(37) $$\omega_{S,t|t-1}^{i}\left(\ell \right)=\frac{p_{s,t|t-1}\left(x_{t-1|t-1}^{i}\left(\ell \right),\ell \right)\omega_{t-1|t-1}^{i}\left(\ell \right)}{\eta_{S,t|t-1}^{\ell }}$$

(38) $$\eta_{S,t|t-1}^{\ell }=\sum_{i=1}^{J_{t-1|t-1}^{\ell }}p_{s,t|t-1}\left(x_{t-1|t-1}^{i}\left(\ell \right),\ell \right)\omega_{t-1|t-1}^{i}\left(\ell \right)$$

(39) $$x_{S,t|t-1}^{i}\left(\ell \right)∼f_{t|t-1}\left(\cdot |\left(x_{t-1|t-1}^{i}\left(\ell \right),\ell \right)\right)$$

where $f_{t|t-1}$ is chosen as the importance sampling density.

**Update**: Denoting the predicted density as $\pi_{t|t-1}={\left\{\left(r_{t|t-1}^{\ell },p_{t|t-1}^{\ell }\right)\right\}}_{\ell \in L_{1:t}}$, it can be written as the form of (6) given by ${\left\{\left(\omega_{t|t-1}\left(I_{+}\right),p_{t|t-1}^{X}\right)\right\}}_{I_{+}\subset L_{1:t}}$, where $\omega_{t|t-1}\left(I_{+}\right)$ is denoted by (7) and $I_{+}=L\left(X\right)$. $p_{t|t-1}^{\ell }$ is represented by a set of weighted particles ${\left\{\left(\omega_{t|t-1}^{i}\left(\ell \right),x_{t|t-1}^{i}\left(\ell \right)\right)\right\}}_{i=1}^{J_{t|t-1}^{\ell }}$. The K-shortest paths algorithm [14] is used to truncate the predicted multi-target density in order to reduce the number of hypotheses. The multi-target posterior density given in (24) is denoted by ${\left\{\left(\omega_{t|t}^{\left(I_{+},\theta \right)}\left(Z\right),{\left[p_{t|t}^{\theta }\right]}^{X}\right)\right\}}_{\left(I_{+},\theta \right)\in F\left(L_{1:t}\right)\times \Theta_{I_{+}}}$. We compute $p_{t|t}^{\theta }\left(x,\ell \right)$ and $\omega_{t|t}^{\left(I_{+},\theta \right)}$ as follows.

$p_{t|t}^{\theta }\left(x,\ell \right)$ can also be represented by a set of particles ${\left\{\left(\omega_{t|t}^{i,\theta \left(\ell \right)},x_{t|t}^{i,\theta (\ell )}\left(\ell \right)\right)\right\}}_{i=1}^{J_{t|t-1}^{\ell }}$ where

(40) $$\omega_{t|t}^{i,\theta \left(\ell \right)}=\frac{\omega_{t|t-1}^{i}\left(\ell \right)\psi_{Z}\left(x_{t|t-1}^{i}\left(\ell \right),\ell ;\theta \right)}{\eta_{Z}^{\theta }\left(\ell \right)}$$

(41) $$x_{t|t}^{i,\theta (\ell )}\left(\ell \right)=x_{t|t-1}^{i}\left(\ell \right)$$

and

(42) $$\eta_{Z}^{\theta }\left(\ell \right)=\sum_{i=1}^{J_{t|t-1}^{\ell }}\omega_{t|t-1}^{i}\left(\ell \right)\psi_{Z}\left(x_{t|t-1}^{i}\left(\ell \right),\ell ;\theta \right)$$

(43) $$\psi_{Z}\left(x_{t|t-1}^{i}\left(\ell \right),\ell ;\theta \right)=\left\{\begin{array}{c} \frac{p_{D}\left(x_{t|t-1}^{i}\left(\ell \right),\ell \right)g\left(z_{\theta \left(\ell \right)}|x_{t|t-1}^{i}\left(\ell \right),\ell \right)}{\kappa \left(z_{\theta \left(\ell \right)}\right)},\theta \left(\ell \right)>0\\ 1-p_{D}\left(x_{t|t-1}^{i}\left(\ell \right),\ell \right),\theta \left(\ell \right)=0 \end{array}\right.$$

The weight $\omega_{t|t}^{\left(I_{+},\theta \right)}$ of the hypothesis $\left(I_{+},\theta \right)$ is given as

(44) $$\omega_{t|t}^{\left(I_{+},\theta \right)}\left(Z\right)=\frac{{\tilde{\omega }}_{t|t}^{\left(I_{+},\theta \right)}\left(Z\right)}{\sum_{\left(I_{+},\theta \right)\in F\left(L_{1:t}\right)\times \Theta_{I_{+}}}{\tilde{\omega }}_{t|t}^{\left(I_{+},\theta \right)}\left(Z\right)}$$

(45) $${\tilde{\omega }}_{t|t}^{\left(I_{+},\theta \right)}\left(Z\right)=\omega_{t|t-1}\left(I_{+}\right){\left[\eta_{Z}^{\theta }\right]}^{I_{+}}$$

where θ is an association function and $\theta \in \Theta_{I_{+}}$. $\left(I_{+},\theta \right)$ is a hypothesis and $\left(I_{+},\theta \right)\in F\left(L_{1:t}\right)\times \Theta_{I_{+}}$. To avoid computing all the hypotheses and their weights, we only reserve a specific number $T_{h}$ of largest weights and the ranked optimal assignment algorithm [14] is applied to truncate the multi-target posterior density.

The multi-target posterior density of the forward LMB filtering matches the first-order moment of (24) which can be denoted as an LMB with the parameter set $\pi_{t|t}\approx {\left\{\left(r_{t|t}^{\ell },p_{t|t}^{\ell }\right)\right\}}_{\ell \in L_{1:t}}$ where $r_{t|t}^{\ell }$ is given by (30) and $p_{t|t}^{\ell }$ is represented by a set of weighted particles ${\left\{\left(\omega_{t|t}^{j}\left(\ell \right),x_{t|t}^{j}\left(\ell \right)\right)\right\}}_{j=1}^{J_{t|t}^{\ell }}$ with

(46) $$\omega_{t|t}^{j}\left(\ell \right)=\frac{\omega_{t|t}^{\left(I_{+},\theta \right)}\left(Z\right)1_{I_{+}}\left(\ell \right)\omega_{t|t}^{i,\theta \left(\ell \right)}}{r_{t|t}^{\ell }}$$

(47) $$x_{t|t}^{j}\left(\ell \right)=x_{t|t}^{i,\theta (\ell )}\left(\ell \right)$$

where $\left(I_{+},\theta \right)\in F\left(L_{1:t}\right)\times \Theta_{I_{+}}$. The number of particles for $p_{t|t}^{\ell }$ increases rapidly and resampling [39] is needed.

**Backward smoothing**: From the forward LMB filtering, the multi-target posterior density is $\pi_{k-1|k-1}={\left\{\left(r_{k-1|k-1}^{\ell },p_{k-1|k-1}^{\ell }\right)\right\}}_{\ell \in L_{1:k-1}}$ at k−1, where $p_{k-1|k-1}^{\ell }$ is approximated by ${\left\{\left(\omega_{k-1|k-1}^{i}\left(\ell \right),x_{k-1|k-1}^{i}\left(\ell \right)\right)\right\}}_{i=1}^{J\left(\ell \right)}$. The predicted multi-target density from k−1 to *k* is an LMB and the existence probability of track *ℓ* is $r_{k|k-1}^{\ell }$ where $\ell \in L_{1:k}$. The multi-target backward smoothed density from *t* to *k* (k≤t) is denoted as $\pi_{k|t}\left(Y\right)={\left\{\left(r_{k|t}^{\ell },p_{k|t}^{\ell }\right)\right\}}_{\ell \in L_{1:k}}$, where $p_{k|t}^{\ell }$ is represented by a set of weighted particles ${\left\{\left(\omega_{k|t}^{j}\left(\ell \right),y_{k|t}^{j}\left(\ell \right)\right)\right\}}_{j=1}^{Q\left(\ell \right)}$.

Proposition 1 implies that the multi-target backward smoothed density from *t* to k−1 is also an LMB given by $\pi_{k-1|t}={\left\{\left(r_{k-1|t}^{\ell },p_{k-1|t}\right)\right\}}_{\ell \in L_{1:k-1}}$ where $r_{k-1|t}^{\ell }$ is calculated by (33) and $p_{k-1|t}^{\ell }$ is represented by a set of weighted particles ${\left\{\left(\omega_{k-1|t}^{i}\left(\ell \right),x_{k-1|t}^{i}\left(\ell \right)\right)\right\}}_{i=1}^{J\left(\ell \right)}$. The detailed formula of $p_{k-1|t}^{\ell }$ is given as

(48) $$\omega_{k-1|t}^{i}\left(\ell \right)=\frac{{\tilde{\omega }}_{k-1|t}^{i}\left(\ell \right)}{\sum_{i=1}^{J\left(\ell \right)}{\tilde{\omega }}_{k-1|t}^{i}\left(\ell \right)}$$

(49) $$\begin{array}{cc} {\tilde{\omega }}_{k-1|t}^{i}\left(\ell \right)= & \frac{\left(1-r_{k|t}^{\ell }\right)\left(1-p_{s,k|k-1}\left(x_{k-1|k-1}^{i}\left(\ell \right),\ell \right)\right)\omega_{k-1|k-1}^{i}\left(\ell \right)}{\left(1-r_{k|k-1}^{\ell }\right)}+\\ \; & \frac{r_{k|t}^{\ell }p_{s,k|k-1}\left(x_{k-1|k-1}^{i}\left(\ell \right),\ell \right)\omega_{k-1|k-1}^{i}\left(\ell \right)}{r_{k|k-1}^{\ell }}\times \\ \; & \sum_{j=1}^{Q\left(\ell \right)}\frac{f_{k|k-1}\left(y_{k|t}^{j}\left(\ell \right)|x_{k-1|k-1}^{i}\left(\ell \right),\ell \right)\omega_{k|t}^{j}\left(\ell \right)}{p_{k|k-1}\left(y_{k|t}^{j}\left(\ell \right),\ell \right)} \end{array}$$

(50) $$x_{k-1|t}^{i}\left(\ell \right)=x_{k-1|k-1}^{i}\left(\ell \right)$$

where $i=1,\cdot \cdot \cdot ,J\left(\ell \right)$ and

(51) $$\begin{array}{cc} \; & p_{k|k-1}\left(y_{k|t}^{j}\left(\ell \right),\ell \right)=\frac{r_{k-1|k-1}^{\ell }}{r_{k|k-1}^{\ell }}\\ \; & \;\;\;\;\;\;\;\;\times \sum_{i=1}^{J\left(\ell \right)}\omega_{k-1|k-1}^{i}\left(\ell \right)p_{s,k|k-1}\left(x_{k-1|k-1}^{i}\left(\ell \right),\ell \right)f_{k|k-1}\left(y_{k|t}^{j}\left(\ell \right)|x_{k-1|k-1}^{i}\left(\ell \right),\ell \right) \end{array}$$

Note that the predicted density (22) cannot be directly used as $p_{k|k-1}\left(\cdot ,\ell \right)$ in (49) for smoothing. Because the forward LMB filtering performs the resampling procedure [39] in each filtering step, the particles of $p_{k|t}\left(\cdot ,\ell \right)$ from $p_{t|t}\left(\cdot ,\ell \right)$ (initial smoothed density) are different from those of $p_{k|k-1}\left(\cdot ,\ell \right)$. We need to estimate $p_{k|k-1}\left(y_{k|t}^{j}\left(\ell \right),\ell \right)$ for each particle $\left(y_{k|t}^{j}\left(\ell \right),\ell \right)$, $j=1,\cdot \cdot \cdot ,Q\left(\ell \right)$ as in (51).

### 4.2. Backward Smoothing and State Extraction

The pseudo code of the proposed backward smoothing algorithm is given in Algorithm 1. The forward filtering is up to *t* and the lag of the backward smoothing is $L_{d}$. We need to store $L_{d}+1$ multi-target posterior densities from $t-L_{d}$ to *t* and $L_{d}$ multi-target predicted densities from $t-L_{d}+1$ to *t* for $L_{d}$-step backward smoothing recursions. The backward smoothed density $\pi_{k|t}\left(Y\right)$ is initialized with $\pi_{t|t}\left(Y\right)$. In the SMC implementation, pruning, truncation and track cleanup are required and the label set varies with the time, so $L_{1:i}$ is replaced with $L_{i|j}$, where $L_{i|j}$ denotes the label set of the corresponded density $\pi_{i|j}\left(\cdot \right)$ and $L_{i|j}\subset L_{1:i}$. Therefore, we have $L_{k-1|t}=L_{k-1|k-1}⋂L_{k|t}$ to represent the label set of backward smoothing at k−1 which eliminates the labels of newborn tracks at *k* and the labels of the pruned tracks in the forward filtering.

Note that, in our approach, resampling [39] can be applied either as the final step of the forward filtering or after the backward smoothing, which lead to insignificant difference in performance. Target states are extracted from the output ${\left\{\left(r_{t-L_{d}|t}^{\ell },p_{t-L_{d}|t}^{\ell }\right)\right\}}_{\ell \in L_{t-L_{d}|t}}$. That is, the target number *N* is first estimated by

(52) $$\hat{N}=\arg\;\;\max_{n}\left\{p\left(n\right)\right\}$$

where p(n) is the cardinality distribution (9). Then $\hat{N}$ tracks with the largest existence probabilities are extracted and the target states are mean of the corresponding probability densities.

**Algorithm 1:** The proposed backward LMB smoothing algorithm. **Input**: lag $L_{d}$ at time *t*, ${\left\{{\left\{\left(r_{k|k}^{\ell },p_{k|k}^{\ell }\right)\right\}}_{\ell \in L_{k|k}}\right\}}_{k=\max\left(t-L_{d},1\right)}^{t}$, ${\left\{{\left\{r_{k|k-1}^{\ell }\right\}}_{\ell \in L_{k|k-1}}\right\}}_{k=\max\left(t-L_{d},1\right)+1}^{t}$; initialize $\pi_{k|t}\left(Y\right)$ with $\pi_{t|t}\left(Y\right)$; for *k*=*t*:-1:max($t-L_{d}$,1)+1       $L_{k-1|t}=L_{k-1|k-1}⋂L_{k|t}$;       for *q* = 1:size($L_{k-1|t}$,2)             compute $r_{k-1|t}^{\ell_{q}}$ according to (33);             for *j*=1:$Q\left(\ell_{q}\right)$                   estimate $p_{k|k-1}\left(y_{k|t}^{j}\left(\ell_{q}\right),\ell_{q}\right)$ according to (51);             end             for *i*=1:$J\left(\ell_{q}\right)$                   compute $\omega_{k-1|t}^{i}\left(\ell_{q}\right)$ according to (48)–(49);                   $x_{k-1|t}^{i}\left(\ell_{q}\right)=x_{k-1|k-1}^{i}\left(\ell_{q}\right)$;             end       end end **Output**: ${\left\{{\left\{\left(r_{k-1|t}^{\ell },p_{k-1|t}^{\ell }\right)\right\}}_{\ell \in L_{k-1|t}}\right\}}_{k=\max\left(t-L_{d},1\right)+1}^{t}$.

### 4.3. Algorithm Complexity

The major computational cost of the LMB smoother is due to backward LMB smoothing. The computational complexity of the backward LMB smoothing is $O\left(L_{d}NL^{2}\right)$ which can be obtained from the four for-loops of Algorithm 1, where *N* is the number of tracks (or targets) and *L* is the number of particles per track. The computational complexity of the innermost two parallel for-loops is $O\left(L^{2}\right)$, so the computational complexity of the backward LMB smoothing is $O\left(L_{d}NL^{2}\right)$ multiplying by the outermost two for-loops.

The structures of backward smoothing for PHD smoother [17] and MeMBer smoother [27] are similar to that of LMB smoother, and the difference is that the particles of all tracks are used for backward smoothing for PHD smoother and MeMBer smoother while the particles of each track are used for their respective backward smoothing in our LMB smoother. We can obtain that the major computational costs for the PHD smoother (Here, we consider the classic SMC implementation [17] instead of the fast SMC implementation [23]) and the MeMBer smoother are both approximately to $o(L_{d}N^{2}L^{2})$. Therefore, the computational complexity of the proposed LMB smoother is lower than those of PHD smoother [17] and MeMBer smoother [27].

## 5. Simulation Result

A nearly constant turn (NCT) model is considered which has varying turn rate together with noisy range and azimuth measurements [12,13]. The state of the target with label *ℓ* at *t* is denoted as $x_{t}=\left(x_{t},\ell \right)$, where $x_{t}={\left[{\tilde{x}}_{t}^{T},w_{t}\right]}^{T}$, ${\tilde{x}}_{t}={\left[p_{x,t},{\dot{p}}_{x,t},p_{y,t},{\dot{p}}_{y,t}\right]}^{T}$ denotes the planar position and velocity of the target, and $w_{t}$ denotes the turn rate. The NCT model can be written as

(53) $${\tilde{x}}_{t}=F\left(w_{t-1}\right){\tilde{x}}_{t-1}+GW_{t-1}$$

(54) $$w_{t}=w_{t-1}+u_{t-1}T$$

where $W_{t-1}$ and $u_{t-1}$ are the state noises of velocity and turn rate, respectively. $W_{t-1}∼N\left(W;0,\sigma_{W}^{2}I_{2}\right)$ and $u_{t-1}∼N\left(u;0,\sigma_{u}^{2}I_{1}\right)$ where $N\left(\cdot ;m_{N},P_{N}\right)$ denotes a Gaussian density with mean $m_{N}$ and variance $P_{N}$, and $I_{i}$ represents an *i* order identity matrix. The noise of velocity uses $\sigma_{W}=5$ m/s${}^{2}$ and the noise of turn rate uses $\sigma_{u}=\pi /180$ rad/s${}^{2}$. The state transition matrix and noise transition matrix are given as follows, respectively,

(55) $$F\left(w\right)=\left[\begin{array}{cccc} 1 & \frac{\sin wT}{w} & 0 & -\frac{1-\cos wT}{w}\\ 0 & \cos wT & 0 & -\sin wT\\ 0 & \frac{1-\cos wT}{w} & 1 & \frac{\sin wT}{w}\\ 0 & \sin wT & 0 & \cos wT \end{array}\right],G=\left[\begin{array}{cc} \frac{T^{2}}{2} & 0\\ T & 0\\ 0 & \frac{T^{2}}{2}\\ 0 & T \end{array}\right]$$

The state transition model is denoted by (53) and (54). The sampling interval is T=1 s. The target survival probability is $P_{s}=0.99$. Newborn targets of each time step is an LMB RFS which can be represented as $\pi_{B,t}={\left\{\left(r_{B,t}^{\ell },p_{B,t}^{\ell }\right)\right\}}_{\ell \in B}$ where $B=\left\{\ell_{1},\ell_{2}\right\}$, $r_{B,t}^{\ell_{1}}=0.02$, $r_{B,t}^{\ell_{2}}=0.03$, and $p_{B,t}^{\ell_{i}}∼N\left(\cdot ;m_{B,t}^{\ell_{i}},P_{B}\right)$. The mean of track $\ell_{1}$ is $m_{B,k}^{\ell_{1}}={\left[-1500,\;0,\;250,\;0,\;0\;\right]}^{T}$, the mean of track $\ell_{2}$ is $m_{B,k}^{\ell_{2}}={\left[1000,\;0,\;1500,\;0,\;0\right]}^{T}$, and the variances of both are $P_{B}=diag{\left(50,50,50,50,6\pi /180\right)}^{2}$. The unit of position $p_{x},p_{y}$ is *m*, the unit of velocity ${\dot{p}}_{x},{\dot{p}}_{y}$ is m/s, and the unit of the turn rate *w* is rad/s.

The measurement equation is

(56) $$z_{t}={\left[\sqrt{p_{x,t}^{2}+p_{y,t}^{2}},\arctan\left(\frac{p_{y,t}}{p_{x,t}}\right)\right]}^{T}+\varepsilon_{t}$$

where the measurement noise is $\varepsilon_{t}∼N\left(\cdot ;0,P_{\varepsilon }\right)$ with $P_{\varepsilon }=diag\left(\sigma_{r}^{2},\sigma_{\theta }^{2}\right)$, $\sigma_{r}=10$ m, and $\sigma_{\theta }=2\pi /180$ rad. The observation region is $\left[\;0,2000\right]$ m$\times \left[\;0,\pi \right]$ rad with the detection probability $p_{D}=0.98$. The density of the Poisson clutter is $\kappa \left(\cdot \right)=10/\left(2000\pi \right)\;$ as the average number of clutter measurements is 10 per scan.

The number of particles per hypothesized track is set to 1000. We prune the tracks with a weight smaller than $P_{T}=10^{-4}$. The lag of the smoother is $L_{d}=3$. The OSPA metric [40] with cut-off parameter c=100 m and order parameter p=1 is used. We compare the proposed LMB smoother with the LMB filter [15], the PHD filter [9], the PHD smoother [17], the CBMeMBer filter [12] and the MeMBer smoother [27] over 100 Monte Carlo trials.

**Figure 3:**
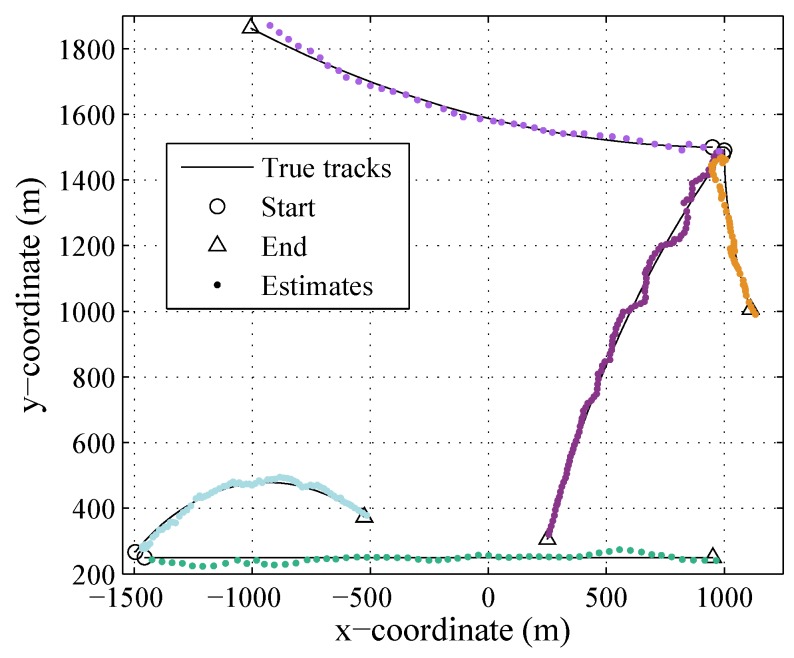
The true and estimated trajectories of targets. Different trajectories estimated by the LMB smoother are denoted by different color dots where ‘∘’ denotes the initiations and ‘Δ’ denotes the terminations of the trajectories.

**Figure 4:**
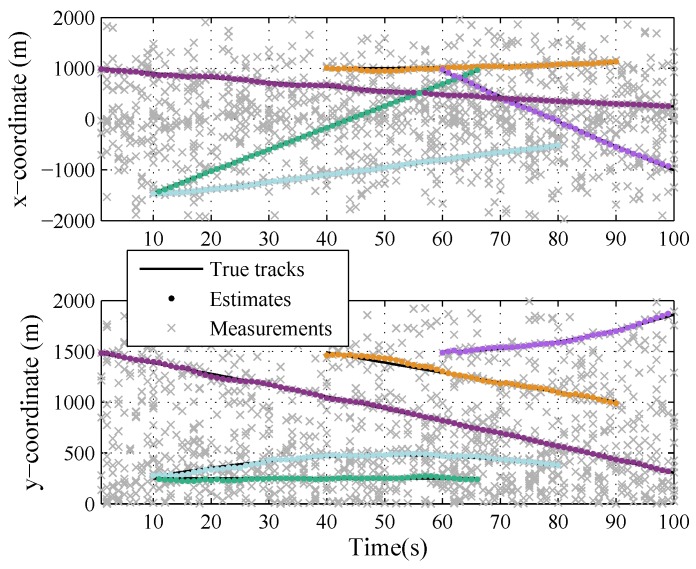
The true and estimated trajectories of targets in x and y coordinates, respectively.

Figure 3 and Figure 4 show the results of the LMB smoother in one trial, in the plane and in *x* and *y* coordinates over time, respectively. The number of targets changes over time due to target birth or death, and there are at most five targets in the scenario. The track of each target is smoothing.

**Figure 5:**
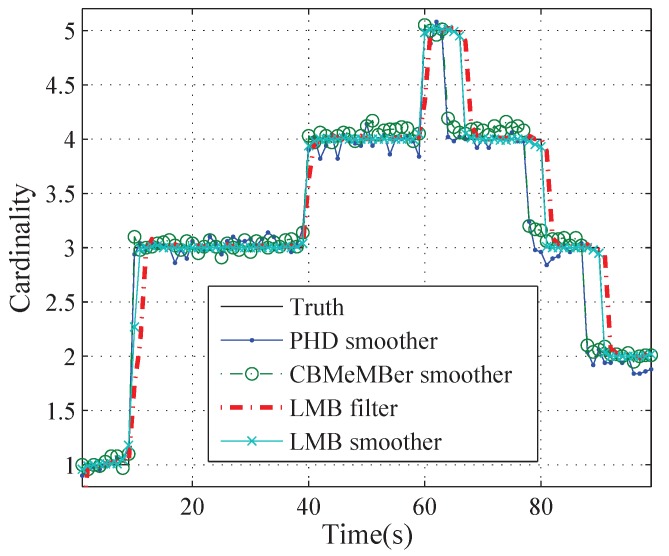
The true and estimated cardinalities of the PHD smoother, the MeMBer smoother, the LMB filter and the proposed LMB smoother.

**Figure 6:**
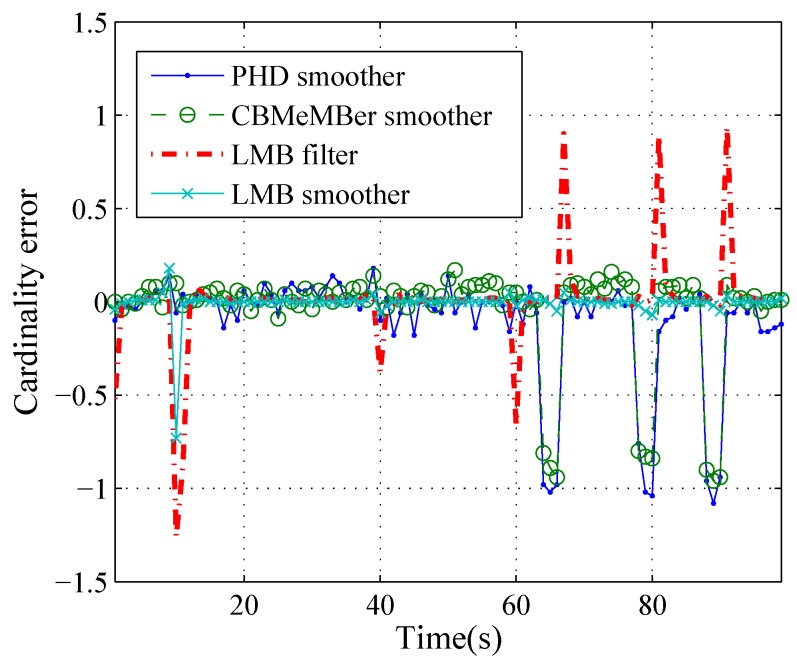
The estimated cardinality errors over time.

**Figure 7:**
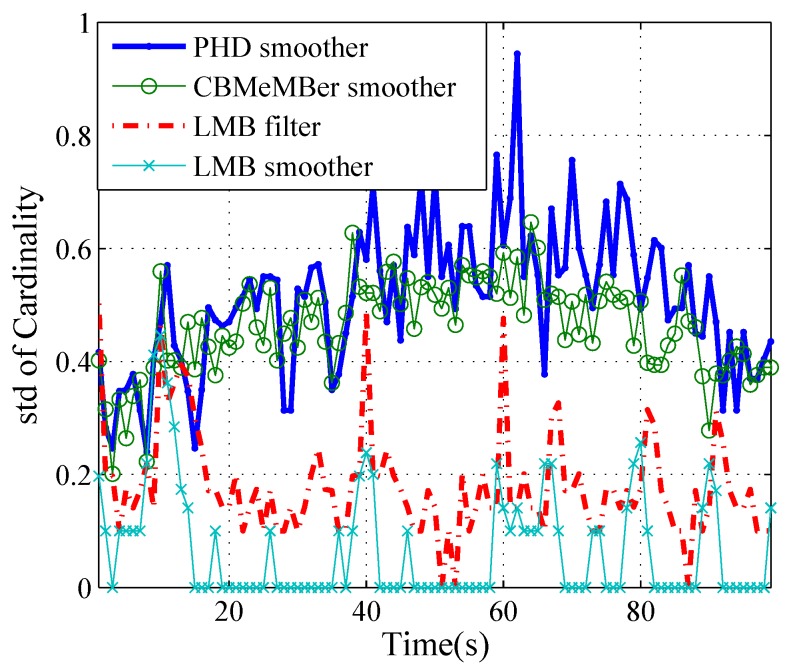
The estimated standard deviations of the cardinality over time.

More specifically, Figure 5, Figure 6 and Figure 7 show the cardinality estimation for different methods over 100 Monte Carlo trials. We can see from Figure 5 that the estimated cardinality mean converges to the true cardinality most of time for all methods. Figure 6 shows the errors of the cardinality mean which is given by the estimated cardinality mean minus the true cardinality. At k=1 s, 10 s, 40 s and 60 s, there is one or two newborn targets at each time step and the cardinality errors are negative for the LMB filter, because of newborn target detection delays. At k=67 s, 81 s and 91 s, one target disappears and the cardinality errors are positive for the LMB filter, because of the detection delay of the target death. At *k* = 64∼66 s, 78∼80 s and 88∼90 s, the cardinality mean errors are negative for PHD and MeMBer smoother, because of premature deaths of targets. Figure 7 shows the standard deviation of the cardinality estimate for different methods. The standard deviations of the PHD and the MeMBer smoother are larger than those of LMB filter and LMB smoother. In short, the LMB smoother can accurately estimate the cardinality (except for a detection delay at k=10 s) and yields the best accuracy.

**Figure 8:**
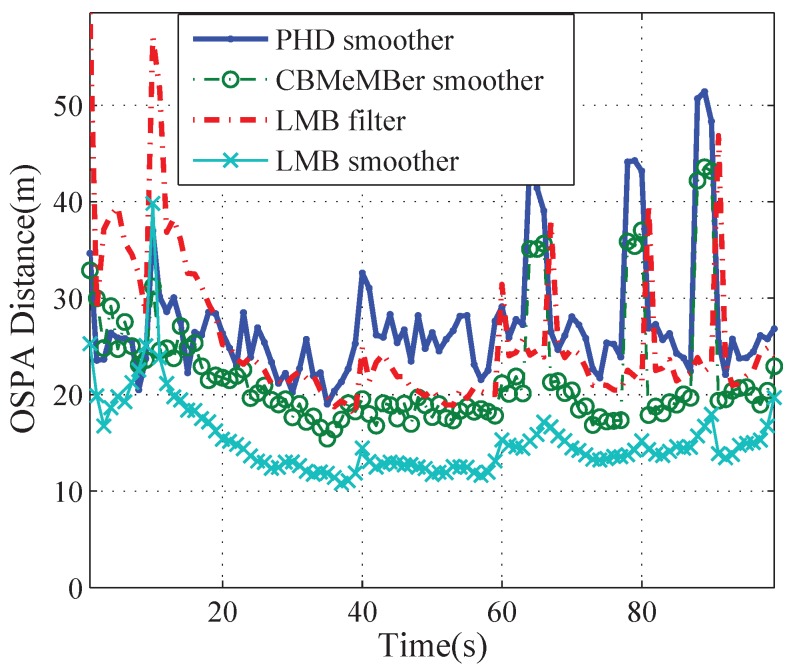
OSPA errors yielded by different methods.

Figure 8 shows the average OSPA errors of the PHD smoother, the MeMBer smoother, the LMB filter, and the LMB smoother with 100 Monte Carlo trials. It can be seen that the OSPA of the LMB smoother is less than those of the other methods almost all the time. We also give the average OSPA errors of different methods in Table 1. It can be seen that all three kinds of smoothers can effectively reduce OSPA localization components as compared with the corresponding filters. However, the PHD smoother and the MeMBer smoother do not necessarily reduce the OSPA cardinality components, whereas the proposed LMB smoother can improve the cardinality estimation significantly.

**Figure 9:**
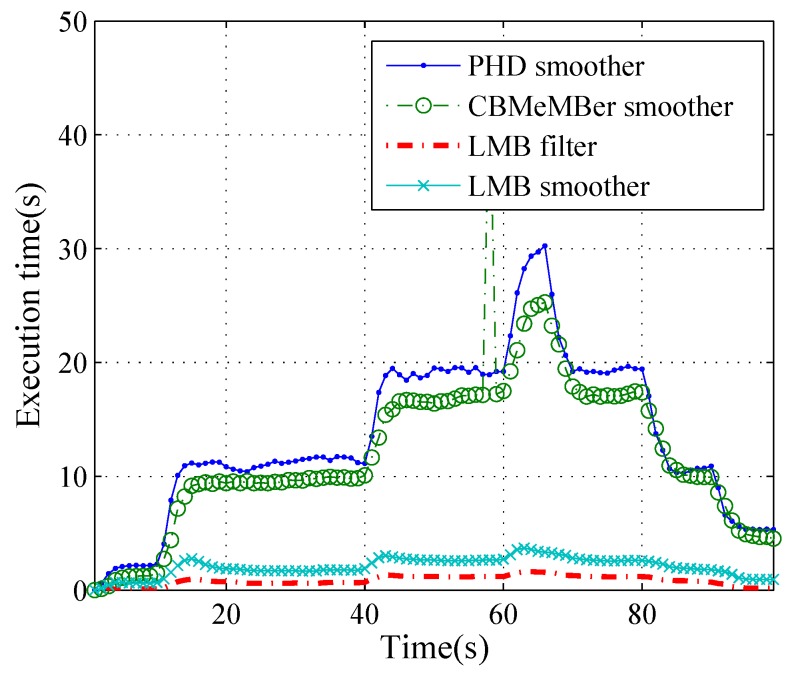
The average execution time of each time step for different methods.

Figure 9 shows the average execution time of different methods over 100 Monte Carlo trials. Summing the time of every time step, we get the times per simulation for the PHD smoother, the MeMBer smoother, the LMB filter, and the LMB smoother which are 1341 s, 1537 s, 156 s and 356 s, respectively. The proposed LMB smoother have a higher computational complexity than the LMB filter, but obviously lower than the PHD smoother and the MeMBer smoother. The results comply with our theoretical analysis.

## 6. Conclusions

The paper derives a computationally efficient forward–backward LMB smoother which is closed under the backward smoothing operation and has the advantages for maintaining the independence of different tracks and their track outputs. Both numerical and simulation analyses have demonstrated that the proposed LMB smoother can effectively improve the tracking performance as compared to the PHD smoother, the MeMBer smoother and the LMB filter and have a lower computational complexity as compared with the PHD smoother and the MeMBer smoother. We should point out that our smoother cannot solve the problem of track fragmentation [34] when the label of a track changes before the track ends. It is our future work to investigate the curve/track fitting approach [19,35,36] for improving the continuity and smoothness of the estimated tracks.

## Figures and Tables

**Table 1:** Average OSPA miss distances of different methods for all time.

Method	Total	Localization	Cardinality
	OSPA (m)	Component (m)	Component (m)
PHD filter	34.3821	26.4094	7.9727
PHD smoother	27.3199	18.6526	8.6673
CBMeMBer filter	30.2842	22.3566	7.9276
MeMBer smoother	22.0721	14.6664	7.4056
LMB filter	25.6800	22.6574	3.0226
LMB smoother	15.1762	14.4932	0.6830

## Appendix A

**Proof of** **Lemma 1.** If $X_{a}$ and $X_{b}$ are both LMB RFSs and $L_{a}⋂L_{b}=⌀$, $X=X_{a}⋃X_{b}$ is an LMB RFS. The conclusion can be obtained from the fundamental convolution theorem [2] and also from the independence of the existence probabilities and the densities of different tracks for LMB RFSs [31]. The product of $\pi_{a}\left(X_{a}\right)$ and $\pi_{b}\left(X_{b}\right)$ can be denoted as

(A1) $$\begin{array}{cc} \pi_{a}\left(X_{a}\right)\pi_{b}\left(X_{b}\right)= & \Delta \left(X_{a}⋃X_{b}\right)\left(\prod_{\ell \in L_{a}⋃L_{b}}\left(1-r^{\ell }\right)\right)\times \\ \; & \left(\prod_{\ell \in L\left(X_{a}⋃X_{b}\right)}\frac{1_{L_{a}⋃L_{b}}\left(\ell \right)r^{\ell }}{\left(1-r^{\ell }\right)}\right){\left[p\left(x,\ell \right)\right]}^{X_{a}⋃X_{b}}\\ = & \Delta \left(X\right)\left(\prod_{\ell \in L}\left(1-r^{\ell }\right)\prod_{\ell \in L\left(X\right)}\frac{1_{L}\left(\ell \right)r^{\ell }}{\left(1-r^{\ell }\right)}\right){\left[p\left(x,\ell \right)\right]}^{X}\\ = & \pi \left(X\right) \end{array}$$

where $L=L_{a}⋃L_{b}$ represents the label space of X. Note that the computation of (A1) is reversible. That is, the union of multiple LMB RFSs on the disjoint subspaces can compose a single LMB RFS on the joint space and vice versa. □

## Appendix B

**Proof of** **Proposition 1.** The proof of Proposition 1 can be summarized as: Firstly, we can decompose the set integral of the backward smoothing recursion (13) into two parts as (A4): One relates to the survived targets, and the other relates to the newborn targets. Then, the second part for the newborn targets is equal to 1 as in (A7). We can conclude that newborn targets cannot transmit to the time before birth, and the set integral of (13) equals to the set integral of the first part for the survived targets. Finally, we prove that the set integral of the first part for the survived targets is an LMB. From the assumptions, $\pi_{k|t}\left(Y\right)$, $\pi_{k|k-1}\left(Y\right)$ and $\pi_{k-1|k-1}\left(X\right)$ are all LMB distributions. $\pi_{k|t}\left(Y\right)$ is denoted as

(A2) $$\pi_{k|t}\left(Y\right)=\Delta \left(Y\right)\prod_{\ell \in L_{1:k}}\left(1-r_{k|t}^{\ell }\right)\prod_{\ell \in L\left(Y\right)}\frac{1_{L_{1:k}}\left(\ell \right)r_{k|t}^{\ell }p_{k|t}\left(y,\ell \right)}{\left(1-r_{k|t}^{\ell }\right)}$$

The label space of $\pi_{k|k-1}\left(Y\right)$ is $L_{1:k}$. $\pi_{k|t}\left(Y\right)$ is initialized with $\pi_{t|t}\left(Y\right)$ and its label space is $L_{1:k}$ when k=t. $\pi_{k|t}\left(Y\right)$ keeps the same label space with $\pi_{k|k-1}\left(Y\right)$ at all other times as to be explained after (A7). The newborn targets can be denoted by an LMB RFS $\pi_{B,k|k-1}={\left\{\left(r_{B,k|k-1}^{\ell },p_{B,k|k-1}^{\ell }\right)\right\}}_{\ell \in L_{k}}$. The multi-target transition density $f_{k|k-1}\left(Y|X\right)$ can be denoted by (16). Let $Y=Y^{+}⋃Y^{-}$. $Y^{+}$ denotes the newborn targets at *k* and $L\left(Y^{+}\right)\subseteq L_{k}$. $Y^{-}$ denotes the surviving targets from k−1 to *k* and $L\left(Y^{-}\right)\subseteq L_{1:k-1}$. The backward smoothing recursion (13) can be reformulated as

(A3) $$\pi_{k-1|t}\left(X\right)=\pi_{k-1|k-1}\left(X\right)\int f_{B,k|k-1}\left(Y^{+}\right)f_{s,k|k-1}\left(Y^{-}|X\right)\frac{\pi_{k|t}\left(Y\right)}{\pi_{k|k-1}\left(Y\right)}\delta Y$$

(A4) $$=\underset{\mathrm{Survived}\mathrm{targets}}{\underset{︸}{\pi_{k-1|k-1}\left(X\right)\int f_{s,k|k-1}\left(Y^{-}|X\right)\frac{\pi_{k|t}^{-}\left(Y^{-}\right)}{\pi_{k|k-1}^{-}\left(Y^{-}\right)}\delta Y^{-}}}\underset{\mathrm{Born}\mathrm{targets}}{\underset{︸}{\int f_{B,k|k-1}\left(Y^{+}\right)\frac{\pi_{k|t}^{+}\left(Y^{+}\right)}{\pi_{k|k-1}^{+}\left(Y^{+}\right)}\delta Y^{+}}}$$

where

(A5) $$\pi_{k|t}\left(Y\right)=\pi_{k|t}^{-}\left(Y^{-}\right)\pi_{k|t}^{+}\left(Y^{+}\right)$$

(A6) $$\pi_{k|k-1}\left(Y\right)=\pi_{k|k-1}^{-}\left(Y^{-}\right)\pi_{k|k-1}^{+}\left(Y^{+}\right)$$

The decomposition of $\pi_{k|t}\left(Y\right)$ by (A5) as well as $\pi_{k|k-1}\left(Y\right)$ by (A6) complies with Lemma 1. The derivation from (A3) to (A4) also applies the Proposition in Section 3.5.3 of [2] that the single set integral on the joint space can be denoted as a multiple set integral on the disjoint subspaces. The Formula (A4) consists of two parts: One relates to X which involves the smoothing of survived targets, and the other is the smoothing of the newborn targets. As $f_{B,k|k-1}\left(Y^{+}\right)=\pi_{k|k-1}^{+}\left(Y^{+}\right)$, the second part of (A4) is equal to 1 and the result can be obtained from

(A7) $$\int f_{B,k|k-1}\left(Y^{+}\right)\frac{\pi_{k|t}^{+}\left(Y^{+}\right)}{\pi_{k|k-1}^{+}\left(Y^{+}\right)}\delta Y^{+}=\int \pi_{k|t}^{+}\left(Y^{+}\right)\delta Y^{+}=1$$

The Formula (A7) can be explained as follows: Newborn targets cannot transmit to the time before birth, i.e., if a target is born at *k*, it cannot be alive at k−1 via backward LMB smoothing. Furthermore, $\pi_{k|t}\left(Y\right)$ cannot have the targets born after *k*, and so $\pi_{k|t}\left(Y\right)$ and $\pi_{k|k-1}\left(Y\right)$ have the same label space $L_{1:k}$ which is the union of the label space at k−1 and the label space of newborn targets at *k*. Combining (35)–(36) and (A7), the Formula (A4) can be further written as

(A8) $$\begin{array}{ccc} \pi_{k-1|t}\left(X\right) & = & \pi_{k-1|k-1}\left(X\right)\int f_{s,k|k-1}\left(Y^{-}|X\right)\frac{\pi_{k|t}^{-}\left(Y^{-}\right)}{\pi_{k|k-1}^{-}\left(Y^{-}\right)}\delta Y^{-}\\ & = & \pi_{k-1|k-1}\left(X\right)\Delta \left(X\right){\left(\frac{1-r_{k|t}^{\ell }}{1-r_{k|k-1}^{\ell }}\right)}^{L_{1:k-1}}{\left(1-p_{s,k|k-1}\left(x,\ell \right)\right)}^{X}\int \Delta \left(Y^{-}\right)\\ & & \times 1_{L\left(X\right)}\left(L\left(Y^{-}\right)\right){\left(\frac{\left(1-r_{k|k-1}^{\ell }\right)p_{s,k|k-1}\left(x,\ell \right)r_{k|t}^{\ell }f_{k|k-1}\left(y|x,\ell \right)p_{k|t}\left(y,\ell \right)}{\left(1-p_{s,k|k-1}\left(x,\ell \right)\right)\left(1-r_{k|t}^{\ell }\right)r_{k|k-1}^{\ell }p_{k|k-1}\left(y,\ell \right)}\right)}^{Y^{-}}\delta Y^{-}\\ & = & \pi_{k-1|k-1}\left(X\right){\left(\frac{1-r_{k|t}^{\ell }}{1-r_{k|k-1}^{\ell }}\right)}^{L_{1:k-1}-L\left(X\right)}{\left(\alpha_{s,k|t}\left(x,\ell \right)\right)}^{X}\int \Delta \left(Y^{-}\right)\\ & & \times 1_{L\left(X\right)}\left(L\left(Y^{-}\right)\right){\left(\frac{\beta_{s,k|t}\left(x,\ell \right)}{\alpha_{s,k|t}\left(x,\ell \right)}\right)}^{L\left(Y^{-}\right)}{\left(\frac{f_{k|k-1}\left(y|x,\ell \right)p_{k|t}\left(y,\ell \right)}{p_{k|k-1}\left(y,\ell \right)}\right)}^{Y^{-}}\delta Y^{-} \end{array}$$

Applying Lemma 3 of [13] (or Lemma 1 in Section 15.5.1 of [2]) and the power-functional identity in Section 3.7 of [2], we have

(A9) $$\begin{array}{cc} \pi_{k-1|t}\left(X\right)= & \pi_{k-1|k-1}\left(X\right){\left(\frac{1-r_{k|t}^{\ell }}{1-r_{k|k-1}^{\ell }}\right)}^{L_{1:k-1}-L\left(X\right)}{\left(\alpha_{s,k|t}\left(x,\ell \right)\right)}^{X}\\ \; & \;\;\;\;\;\;\;\;\times \sum_{L\left(Y^{-}\right)\subseteq L\left(X\right)}\prod_{\ell \in L\left(Y^{-}\right)}\int \frac{\beta_{s,k|t}\left(x,\ell \right)f_{k|k-1}\left(y|x,\ell \right)p_{k|t}\left(y,\ell \right)}{\alpha_{s,k|t}\left(x,\ell \right)p_{k|k-1}\left(y,\ell \right)}dy\\ \; & =\pi_{k-1|k-1}\left(X\right){\left(\frac{1-r_{k|t}^{\ell }}{1-r_{k|k-1}^{\ell }}\right)}^{L_{1:k-1}-L\left(X\right)}{\left(\alpha_{s,k|t}\left(x,\ell \right)\right)}^{X}\\ \; & \;\;\;\;\;\;\;\;\times \prod_{\ell \in L\left(X\right)}\left(1+\int \frac{\beta_{s,k|t}\left(x,\ell \right)f_{k|k-1}\left(y|x,\ell \right)p_{k|t}\left(y,\ell \right)}{\alpha_{s,k|t}\left(x,\ell \right)p_{k|k-1}\left(y,\ell \right)}dy\right)\\ \; & =\pi_{k-1|k-1}\left(X\right){\left(\frac{1-r_{k|t}^{\ell }}{1-r_{k|k-1}^{\ell }}\right)}^{L_{1:k-1}-L\left(X\right)}\\ \; & \;\;\;\;\;\;\;\;\times \prod_{\ell \in L\left(X\right)}\left(\alpha_{s,k|t}\left(x,\ell \right)+\int \beta_{s,k|t}\left(x,\ell \right)\frac{f_{k|k-1}\left(y|x,\ell \right)p_{k|t}\left(y,\ell \right)}{p_{k|k-1}\left(y,\ell \right)}dy\right) \end{array}$$

Substituting $\pi_{k-1|k-1}\left(X\right)$ into (A9) yields

(A10) $$\begin{array}{cc} \pi_{k-1|t}\left(X\right)= & \Delta \left(X\right)\prod_{\ell \in L_{1:k-1}-L\left(X\right)}\frac{\left(1-r_{k-1|k-1}^{\ell }\right)\left(1-r_{k|t}^{\ell }\right)}{\left(1-r_{k|k-1}^{\ell }\right)}\prod_{\ell \in L\left(X\right)}\left(1_{L_{1:k-1}}\left(\ell \right)r_{k-1|k-1}^{\ell }\right.\\ \; & \;\;\;\;\;\;\;\;\times \left.p_{k-1|k-1}\left(x,\ell \right)\left(\alpha_{s,k|t}\left(x,\ell \right)+\int \beta_{s,k|t}\left(x,\ell \right)\frac{f_{k|k-1}\left(y|x,\ell \right)p_{k|t}\left(y,\ell \right)}{p_{k|k-1}\left(y,\ell \right)}dy\right)\right) \end{array}$$

Let

(A11) $$\begin{array}{cc} \pi_{k-1|t}\left(x,\ell \right)= & r_{k-1|t}^{\ell }p_{k-1|t}\left(x,\ell \right)\\ = & r_{k-1|k-1}^{\ell }p_{k-1|k-1}\left(x,\ell \right)\left(\alpha_{s,k|t}\left(x,\ell \right)+\int \frac{\beta_{s,k|t}\left(x,\ell \right)f_{k|k-1}\left(y|x,\ell \right)p_{k|t}\left(y,\ell \right)}{p_{k|k-1}\left(y,\ell \right)}dy\right) \end{array}$$

We can obtain that

(A12) $$r_{k-1|t}^{\ell }=\int \pi_{k-1|t}\left(x,\ell \right)dx$$

(A13) $$\begin{array}{cc} \; & p_{k-1|t}\left(x,\ell \right)=\frac{\pi_{k-1|t}\left(x,\ell \right)}{r_{k-1|t}^{\ell }}=\frac{\pi_{k-1|t}\left(x,\ell \right)}{\int \pi_{k-1|t}\left(x,\ell \right)dx} \end{array}$$

From (A12), we can obtain (33) and the proof is further given in Appendix C. We can also obtain (34) by substituting (A11) into (A13). Substituting (A12) and (A13) (or (33) and (34)) into (A10) yields

(A14) $$\begin{array}{cc} \pi_{k-1|t}\left(X\right)= & \Delta \left(X\right)\prod_{\ell \in L_{1:k-1}-L\left(X\right)}\left(1-r_{k-1|t}^{\ell }\right)\prod_{\ell \in L\left(X\right)}\left(1_{L_{1:k-1}}\left(\ell \right)r_{k-1|t}^{\ell }p_{k-1|t}\left(x,\ell \right)\right)\\ = & \Delta \left(X\right)\prod_{\ell \in L_{1:k-1}}\left(1-r_{k-1|t}^{\ell }\right)\prod_{\ell \in L\left(X\right)}\frac{1_{L_{1:k-1}}\left(\ell \right)r_{k-1|t}^{\ell }p_{k-1|t}\left(x,\ell \right)}{\left(1-r_{k-1|t}^{\ell }\right)} \end{array}$$

From (A14), we get the parameter set (32) which indicates that $\pi_{k-1|t}\left(X\right)$ is an LMB where $r_{k-1|t}^{\ell }$ and $p_{k-1|t}\left(x,\ell \right)$ are given in (33) and (34), respectively. □

## Appendix C

**Proof.** **(from (A12) to (33)).** Firstly, it is known that the target $\left(x,\ell \right)$ at *k* is the survived target in (A12), $\ell \in L_{1:k-1}$ and $\ell \notin L_{k}$. This is because newborn targets cannot transmit to the time before birth via smoothing from (61) (that is, $\pi_{k-1/t}\left(X\right)$ cannot have the targets born after k−1). Therefore, we can apply the labeled single target predicted density formula (the same as (20) and (21) for the survived target)

(A15) $$r_{k|k-1}^{\ell }=r_{k-1|k-1}^{\ell }\int p_{s,k|k-1}\left(x,\ell \right)p_{k-1|k-1}\left(x,\ell \right)dx$$

(A16) $$p_{k|k-1}\left(y,\ell \right)=\frac{\int r_{k-1|k-1}^{\ell }p_{k-1|k-1}\left(x,\ell \right)p_{s,k|k-1}\left(x,\ell \right)f_{k|k-1}\left(y|x,\ell \right)dx}{r_{k|k-1}^{\ell }}$$

Then, from the definition of (A11) and (A12), we can obtain

(A17) $$\begin{array}{cc} r_{k-1|t}^{\ell }= & \int \pi_{k-1|t}\left(x,\ell \right)dx\\ = & \int \alpha_{s,k|t}\left(x,\ell \right)r_{k-1|k-1}^{\ell }p_{k-1|k-1}\left(x,\ell \right)dx+\\ \; & \;\int \int \;\frac{r_{k-1|k-1}^{\ell }p_{k-1|k-1}\left(x,\ell \right)\beta_{s,k|t}\left(x,\ell \right)f_{k|k-1}\left(y|x,\ell \right)p_{k|t}\left(y,\ell \right)}{p_{k|k-1}\left(y,\ell \right)}dydx \end{array}$$

where the first term of the right of (A17) can be written as

(A18) $$\begin{array}{cc} \; & \int \alpha_{s,k|t}\left(x,\ell \right)r_{k-1|k-1}^{\ell }p_{k-1|k-1}\left(x,\ell \right)dx\\ \; & =\frac{\left(1-r_{k|t}^{\ell }\right)r_{k-1|k-1}^{\ell }}{\left(1-r_{k|k-1}^{\ell }\right)}\int \left(1-p_{s,k|k-1}\left(x,\ell \right)\right)p_{k-1|k-1}\left(x,\ell \right)dx\\ \; & =\frac{\left(1-r_{k|t}^{\ell }\right)}{\left(1-r_{k|k-1}^{\ell }\right)}\left(r_{k-1|k-1}^{\ell }-r_{k|k-1}^{\ell }\right) \end{array}$$

The second term of the right of (A17) can be written as

(A19) $$\begin{array}{cc} \; & \;\int \int \;\frac{r_{k-1|k-1}^{\ell }p_{k-1|k-1}\left(x,\ell \right)\beta_{s,k|t}\left(x,\ell \right)f_{k|k-1}\left(y|x,\ell \right)p_{k|t}\left(y,\ell \right)}{p_{k|k-1}\left(y,\ell \right)}dydx\\ \; & =\int \frac{\int r_{k-1|k-1}^{\ell }p_{k-1|k-1}\left(x,\ell \right)p_{s,k|k-1}\left(x,\ell \right)f_{k|k-1}\left(y|x,\ell \right)dx}{r_{k|k-1}^{\ell }}\frac{r_{k|t}^{\ell }p_{k|t}\left(y,\ell \right)}{p_{k|k-1}\left(y,\ell \right)}dy\\ \; & =\int r_{k|t}^{\ell }p_{k|t}\left(y,\ell \right)dy\\ \; & =r_{k|t}^{\ell } \end{array}$$

Finally, combining (A17)–(A19) yields

(A20) $$r_{k-1|t}^{\ell }=\frac{\left(1-r_{k|t}^{\ell }\right)}{\left(1-r_{k|k-1}^{\ell }\right)}\left(r_{k-1|k-1}^{\ell }-r_{k|k-1}^{\ell }\right)+r_{k|t}^{\ell }=1-\frac{\left(1-r_{k-1|k-1}^{\ell }\right)\left(1-r_{k|t}^{\ell }\right)}{1-r_{k|k-1}^{\ell }}$$

 □
